# Unveiling the Chemical Composition, Bioactive Profile and Antioxidant Capacity of Dried Egyptian Jew’s Mallow Stems as a Promising Anticancer Agent

**DOI:** 10.3390/molecules29061377

**Published:** 2024-03-20

**Authors:** Marwa Rashad Ali, Huda Hassan Ibrahim, Aziza Ali Salah-Eldin

**Affiliations:** 1 Food Science Department, Faculty of Agriculture, Cairo University, Giza 12613, Egypt; 2 Food Technology Research Institute, Agricultural Research Center, Giza 12619, Egypt; drhudahassan82@gmail.com (H.H.I.); azizaali161@gmail.com (A.A.S.-E.)

**Keywords:** nutritional value, vitamin C, B-complex, chlorophyll, antioxidant activity, DPPH, TPC, flavonoids, HPLC, antitumor

## Abstract

Phytochemicals from waste materials generated by agricultural and industrial processes have become globally significant due to their accessibility and potential effectiveness with few side effects. These compounds have essential implications in both medicine and the economy. Therefore, a quantitative analysis of the phytochemical profile, sugar types, and water-soluble vitamins of dried *Corchorus olitorius* L.“DJMS” extract (dried Jew’s mallow stem) was carried out with HPLC. In addition, the chemical composition, TPC, chlorophyll a and b, beta-carotene, and antioxidant effect using DPPH were investigated. Furthermore, the anticancer activity of the DJMS was evaluated by SRB assay using Huh-7 and MDA-MB-231 cell lines. In the quantitative study, DJMS extract showed a high antioxidant potential (67%) due to its content of bioactive compounds such as TPC (276.37 mg 100 g^−1^) and chlorophyll a and b (20.31, 12.02 mg 100 g^−1^, respectively), as well as some vitamins and minerals such as B-complex (B12; 146.8 mg 100 g^−1^ and vitamin C 6.49 mg 100 g^−1^) and selenium (<0.2 μg kg^−1^). Moreover, the main sugar types found were sucrose and stachyose, which recorded 9.23 and 6.25 mg 100 g^−1^, respectively. Identifying phenolic and flavonoids showed that the major components were ellagic acid (4905.26 μg kg^−1^), ferulic acid (3628.29 μg kg^−1^), chlorogenic acid (3757.08 μg kg^−1^), luteolin—7-*O*-glucoside (4314.48 μg kg^−1^), naringin (4296.94 μg kg^−1^) and apigenin—6—rhamnose—8 glucoside (3078.87 μg kg^−1^). The dried stem extract showed significant MDA-MB-231 inhibition activity and reached 80% at a concentration of 1000 µg/mL of DJMS extract, related to the content of phytochemical components such as isoflavones like genistein (34.96 μg kg^−1^), which had a tremendous anticancer effect. Hence, the stem of Jew’s mallow (which is edible and characterized by its viability and low production cost) possesses the capacity to serve as a pharmaceutical agent for combating cancer owing to its abundance of bioactive components.

## 1. Introduction

Plants have given humans and other organisms food and other health benefits for as long as they have existed [1]. Secondary metabolites, or bioactive chemicals, are present in medicinal plants [2,3,4,5,6]. Secondary metabolites are a class of substances that have been shown to have therapeutic action against several human disorders. This may account for the traditional usage of medicinal plants in treating many diseases. Quantitative assessments of the bioactive compounds in medicinal plants are essential in identifying the matching chemicals in particular plants. Thus, separating these chemicals at large concentrations from the relevant plants is possible [6,7,8]. Particularly in cancer therapy, scientific methods are used to devise treatments for patients. Patients may experience adverse effects from synthetic pharmaceuticals, however. For the treatment of diseases including cancer, Alzheimer’s, and diabetes, therefore, the development and clinical application of pharmaceuticals derived from plant materials are crucial [6,9,10,11]. According to reports, roughly 80% of the global population derives its primary medication from natural products and plant-based medicines [12]. Furthermore, approximately 25% of all medications are sourced from 500 distinct species of medicinal plants [13].

The distinctiveness of the North African and Mediterranean diets is frequently attributed to their abundance of cereal and olive oil. Vegetables contain considerable nutritional value. Mallows and other leafy green vegetables are ubiquitous in Egyptian cuisine. Jew’s mallow, or jute mallow in English, is the term *Corchorus olitorius* called Molokhia. This herb can grow up to 4 feet tall and is either annual or perennial. It is a well-liked green vegetable crop grown in Egypt during the summer. Fresh or dried green leaves add a pleasant flavor to soups and stews [14]. The vegetable is grown for its mucilaginous leaves, which are also used as food; its stem bark, used to make jute fiber; and its seeds, used as flavoring agents [15]. In Southeast Asia, its leaves and roots are also consumed as herbal medicine [16]. It is a genus comprising 50–60 species of annual plants that are members of the Tiliaceae family. These species’ slender, branching stems with mucilaginous leaves are utilized as vegetables in meals to foster community in jute-producing nations [17,18,19,20,21]. Molokhia leaves are edible raw or can be processed (drying, extraction, etc.) to make various goods. Additionally, they can be added to a list of items to enhance and increase their nutritional profile, functional qualities, and organoleptic quality [22,23].

Numerous studies have examined molokhia’s biochemical composition and biological activity. Molokhia leaves contain many beneficial components and are highly valued for their high content of carotenoids, vitamins A, B1, B2, B5, C, and E, folic acid, and minerals (Ca, P, K, Na, Fe) [14]. Molokhia also offers different amounts of healthy fiber and protein [16]. Its pharmacological activities come from phytochemicals such phenols, flavonoids (such as chlorogenic acid, quercetin glycosides, caffeic acid, and isorhamnetin), sterols, glycosides, tannins, saponins, lipids, and fatty acids in plant parts and seeds. These components give the plant anticancer, antioxidant, analgesic, antiviral, antipyretic, diuretic, analgesic, antibacterial, antidiabetic, cardiovascular effects, hepatoprotective, and neuroprotective properties. The leaves have been used in traditional medicine, cuisine, and skin care [6,24,25,26]. Brazilian and other folk medicines use it to treat colitis. Stomatitis, chronic bronchitis, furuncles, abscesses, contusions, gonorrhea, pain, fever, tumors, hemorrhoids, and other painful and inflammatory conditions are treated with plant leaf extract [1,27]. AL Yousef et al. and Yakoub et al. reported that dry oils of leaves and stems include many bioactive components, such as 2, 4-di-tert-butylphenol, and fatty acids, including hexadecenoic and ethyl palmitate. These bioactive substances participate in many biological processes and free radical attenuation, which causes degenerative illnesses. Thus, their bioactive molecules have several beneficial effects, including antioxidant, enzyme inhibitory, anticancer, and antibacterial properties [28,29]. Isuosuo et al. and Atalar et al. showed that *C. olitorius* seeds include calcium, iron, vitamin A, thiamine, riboflavin, nicotinamide, and ascorbic acid. Furthermore, this plant contains cardiac glycosides, phenolic acids, polysaccharides, sterols, and fatty acids, etc. [6,30].

Food manufacturing waste represents a significant problem because of its large quantity due to the expansion of food industry factories and technology. Despite this, it represents substantial economic wealth and is capable of employing young people and idle energies if utilized in an integrated system that includes various economic, social, environmental, and technical aspects, given its content. Waste is an essential element and can be used in multiple forms, thus preserving the environment from pollution produced by accumulating and burning these wastes.

Without a doubt, one of the deadliest threats facing humanity today is cancer. Egypt had 134,632 cancer cases in 2020, with liver (27,895), breast (22,038), bladder (10,655), non-Hodgkin lymphoma (7305), lung (6538), leukemia (5231), and prostate (4767) having the highest incidence. Egypt had 89,042 cancer deaths in 2020, which is considered the highest mortality rate [31]. To our knowledge, no research addresses the chemical composition (carbohydrates, protein, etc.), bioactive profile identification, antioxidant activity, and antiproliferative activities of dried Jew’s mallow stems. Therefore, our study aims to shed light on the diverse array of compounds in botanical components. Thus, the stem of mallow could be considered a hidden treasure and promising cure for different types of cancer, such as liver (Huh-7) and breast (MDA-MB-231).

## 2. Results and Discussion

### 2.1. Chemical Composition

**Figure 1:**
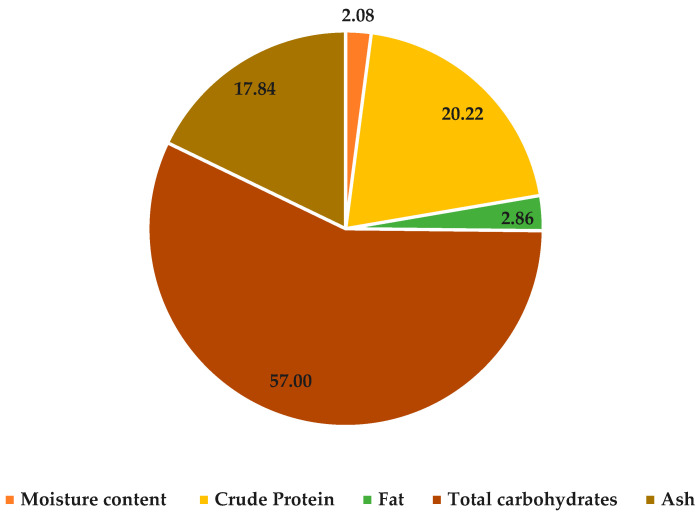
Chemical composition of dried Jew’s mallow stems (g 100 g^−1^).

Technically identified as *Corchorus olitorius* L., mallow is a blossoming plant which has long been used in traditional medical and culinary applications. Diverse parts of the mallow plant have been used for various purposes, but we are interested in the chemical composition of dried mallow stems. Figure 1 displays the primary significance of the nutrient composition and estimated energy value, presented in terms of dry weight. The obtained results show that mallow has a high content of protein, carbohydrate, fat, and ash (20.22, 57.00, 2.86 and 17.84%) in dried stems compared to dried leaves, which ranged between 10.44 and 36.73% for protein, 46.07% for carbohydrate, 1.7 for fat and 7 and 16.13% for ash [31,32,33]. Fat was the least prevalent, with a macronutrient abundance of less than 3%. Therefore, the results reflected the finding that dried stems had a high nutritional value, which could be considered a great supplement, especially protein carbohydrates, as a source of energy and building tissues for different consumer categories, especially children and athletes. Each 100 g of dried stems gives 334.62 Kcal, which agrees with those findings reported by Abbasi et al. [34].

### 2.2. Sugar Content

Regarding sugar composition, DJMS contained stachyose, sucrose, maltose, glucose, rhamnose, galacturonic, xylose, galactose, mannose, fructose, arabinose, mannitol, and ribose as the main sugars (Table 1). The present study describes the sugar composition in dried mallow stems for the first time. Sucrose and stachyose were the most abundant sugars (9.227 and 6.254 g 100 g^−^^1^ of dry weight). Elsayed et al. [35] reported that the types of sugars are analyzed through chromatography; the polysaccharides in the eight *Aloe* species contained 18 saccharides, glucuronic acid, stachyose, galacturonic acid, sucrose, glucose, xylose, galactose, rhamnose, mannose, arabinose, fructose, polyol, mannitol, and sorbitol. The quantitative distribution of these components varied across the different species. Moreover, the polysaccharides produced by alloxan were shown to have antihyperglycemic effects in diabetic rats and an ability to block alpha-glucosidase.

### 2.3. Mineral Content

Otherwise, DJMS is considered a source of minerals because it has a high ash content (17.84 g 100 g^−^^1^). The main elements found in it are Fe, Ca, Mg, P, K, and Se, as shown in Table 1. It could be a great resource for treating malnutrition diseases such as anemia by supplementing different functional foods. Also, some of these elements significantly affect anticancers such as Se. There is evidence suggesting that selenium compounds may possess chemopreventive properties. Research is underway to see if these compounds can also impact established malignancies. In addition, Radomska et al., Frajese et al., and Croci et al. [36,37,38] reported that K ascorbate has been proposed to function as a potassium intracellular transporter and can potentially impede the cell cycle in cancerous cells.

### 2.4. Vitamin Content

The DJMS content of water-soluble vitamins is shown in Table 1. The values of vitamin B complex in dried mallow stems were recorded as 5.62, 67.52, 9.31, 25.68, and 146.80 mg 100 kg^−1^ for B1, B2, B6, B9, and B12, respectively. Meanwhile, vitamin C (ascorbic acid) recorded a low content, reaching 6.49 mg 100 g^−1^ compared to leaves containing 258 mg 100 g^−1^ FW, as documented by Ragasa et al. [39]. Stems of the Jew’s mallow could be considered a source of water-soluble vitamins such as vitamins B and C, especially vitamin B complex, identified for the first time in dried Jew’s mallow stems. Several studies have suggested that vitamin B supplements reduce cancer risks; therefore, one of our research objectives is determining vitamin B [40,41]. In addition, vitamin C is among the most potent antioxidant properties and may be an effective anticancer agent. The metabolomic and epigenetic profiles of cancer cells may be altered by vitamin C, and the elimination of cancer stem cells may result from the vitamin’s activation of ten-eleven translocation (TET) proteins and downregulation of pluripotency factors [42].

### 2.5. Bioactive Profile

The extent of the bioactive profile of the DJMS methanol was assessed. The concentration of total phenolic compounds (TPC) was higher in the dried stems (276.37 mg GAE 100 g^−1^), while Beghdad et al. [43] reported that the TPC in stem extract obtained from *M. sylvestris* L. was lower (217.30 mg GAE 100 g^−1^) than leaves (2412.30 mg GAE 100 g^−1^). Meanwhile, Mouas et al. [44] found that TPC in leaves of *C. olitorius* L. was lower (200 mg GAE 100 g^−1^) than in our study. Also, Ben Yakoub et al. [45] reported that the ethanolic extract of *C. olitorius* leaves showed a low TPC (9.20 mg GAE 100 g^−1^ DW). Other compounds and different types of phenols can explain this variation.

Meanwhile, our study showed that the content of dried Jew’s mallow stem was low in chlorophyll (20.31 and 12.02 mg 100 g^−1^ for chlorophyll a and b, respectively) and Beta-carotene (1.5 mg 100 g^−1^). Upadhyay [46] documented that plant pigments are secondary metabolites that inhibit the proliferation of cancer cells by halting their growth and cell division. These substances hinder biological activities in cancer cells, including signaling pathways, cell cycle regulation, apoptosis, and autophagy. In addition to their anticancer properties, these substances also help regulate excessive blood pressure, obesity, hyperglycemia, and hypercholesterolemia, and address cardiovascular issues.

### 2.6. Identification of Phenolic, Flavonoid and Isoflavone Compounds

Phenolic compounds, a broad family of secondary plant metabolites, are recognized as antioxidants and other dietary reducing agents crucial for the quality of plant-based diets. They maintain the body’s tissues against oxidative stress-related diseases like cancer, inflammation, and coronary heart disease [1]. The phenolic and flavonoid compounds of dried *C. olitorius* methanolic (Table 2) revealed that the stem extract contained high amounts of phenolic compounds, for example, ellagic acid, pyrogallol, chlorogenic acid, ferulic acid, and catechin. Moreover, it had a high number of flavonoids such as apigenin -6-rhamnose-8-glucoside, apigenin-6-arabinose-8-glactoside, naringin, Luteolin 7-glucoside, rosmarinic and quercetin-3-glucoside.

Our results agreed with Khan et al. [47], who reported that *Corchorus* species include chlorogenic acid and quercetin-3-glucoside. Cynarin, chlorogenic acid, quercetin-3-glucoside, and biochanin A are documented to be medicinally critical natural compounds and are found in high concentration in the leaf, stem, and root extracts of the plant, exhibiting significant anticancer, antidiabetic, antibacterial, and anti-inflammatory activities [48]. Chlorogenic acid and related compounds are generally recognized as antioxidants [49]. Otherwise, the predominant isoflavone in the methanolic extract of DJMS was isorhamnetin (5502.00 µg 100 g^−1^) and genistein (59.91 µg 100 g^−1^), as shown in Table 2. The antioxidant properties of isoflavones are generally recognized for their potential benefits to human health. Physical activity can create a disparity between reactive oxygen species (ROS) and antioxidants [50], which indicates that genistein may lower the likelihood of tumor formation [51].

### 2.7. Antioxidant Capacity %

The antioxidant activity achieved a substantial proportion of 67% due to the abundant presence of phenolic compounds and other dietary reducing agents. The findings of our study are consistent with those of Karimi et al. [35], indicating a direct relationship between the number of phenolic compounds and their antioxidant capacity. For example, Lan [49] found that the level of chlorogenic acid in *Flos Lonicerae* extracts correlates with their antioxidant properties. Higher levels of chlorogenic acid enhance the effectiveness of scavenging the DPPH radical, as indicated by the latest results. Moreover, flavonoids are a widespread category of phenolic chemicals that exhibit antioxidant activity in laboratory settings, primarily due to derivatives of naringenin [52]. Naringenin is a prominent flavonoid compound mainly derived from grapefruit and oranges. Naringenin exhibits strong antioxidant properties and can reduce cholesterol levels, prevent cancer, and reduce inflammation [53,54]. Furthermore, it is commonly accepted that isoflavones, such as genistein, positively affect human health due to their antioxidant properties [50]. Genistein, a common soy isoflavone, is an antioxidant that directly donates hydrogen atoms from the hydroxyl group linked to the benzene ring [55,56].

### 2.8. Anticancer Effects of Dried Mallow Stem

**Figure 2:**
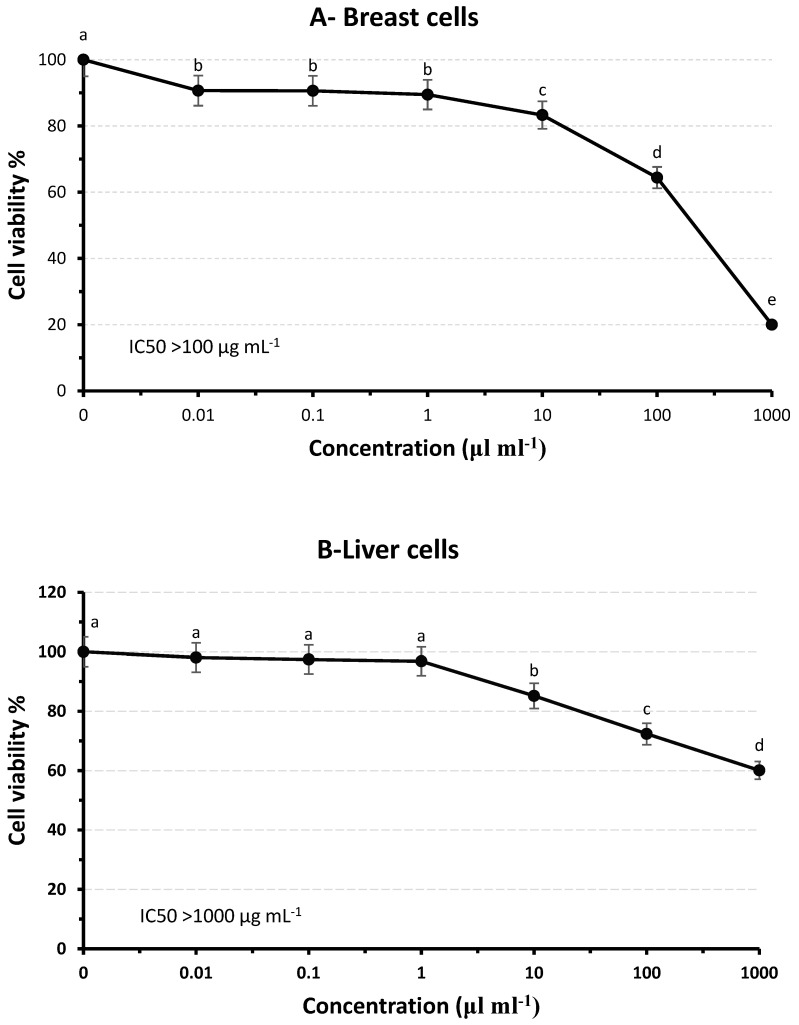
Antiproliferative activity of dried Jew’s mallow stems against MDA-MB-231 (**A**) and Huh-7 (**B**) cancer cells. The experimental values (means and SD for n = 3) with the small letters are significantly different (*p* ≤ 0.05).

To investigate the anticancer effects of dried Jew’s mallow stems, two types of human cancer cell lines (liver “Huh-7” and breast “MDA-MB-231”) were treated with the dried mallow stems. There was evidence that the mallow stem extract suppressed the viability of MDA-MB- 231breast cells by 80% at 1000 µg mL^−1^; meanwhile, it suppressed the viability of Huh-7liver cells by 40% at the same concentration. Also, there was a significant difference between the non-treated cells (*p* < 0.05) and at all low concentrations between 0.01 and 1000 µL/mL. Therefore, the IC50 of MDA-MB-231 is >100 µg mL^−1^; meanwhile, the IC50 for Huh-7 is >1000 µg mL^−1^ (Figure 2). Accordingly, the results reported here agree with those previously reported regarding the highly antiproliferative activity of *C. Olitorius* leaf extract against Caco-2 cell lines and had a low effect on sk-ov-3 cancer cell lines after treatment at the same concentration (250 µg mL^−1^) [6]. In another study, it was stated that the compounds in the *Ocimum basilicum* and *Impatiens walleriana* extracts suppressed cell viability by more than 78% [57]. The anticancer effect may be correlated to the higher content of bioactive compounds, which increase the antioxidant effect, rather than the low concentration of dried mallow stem extract. According to Omoruyi et al. [58], TPC inhibits cancer cell growth, invasion, adhesion, and tube formation due to the substantial content of the bioactive compound of *C. olitorius*, which appears to have considerable antioxidant and antiproliferative activities. Caffeic acids naturally exhibit biological activities such as antioxidant, antimutagenic, anticancer, and anti-obesity properties [55]. In addition, Sarkar and Li [59] showed that genistein, the primary isoflavone in soy, effectively suppresses cancer development in animal models. Therefore, these results suggest that the powder of mallow stem may be preferable as a natural and safe breast cancer therapeutic agent. Moreover, Frajese et al. [37] showed that potassium enhanced the antitumor properties of vitamin C in specific breast cancer cell lines. Furthermore, plant pigments such as carotenoids, anthocyanins, betalains, chlorophyll, and lycopene hinder biological activities in cancer cells, including signaling pathways, cell cycle regulation, apoptosis, and autophagy [46].

## 3. Materials and Methods

### 3.1. Materials and Chemicals

Fresh Jew’s mallow (*Corchorus olitorius* Seady) was purchased from the local market in Giza, Egypt. The species we work on is *Corchorus olitorius* Seady, and it has already been identified by Elwakil et al. [60] under the accession numbers for RbcL and MatK gene in the database of the National Library of Medicine. 

The accession numbers for the RbcL gene, “gene bank: LC732566” [60] and for the MatK gene, “gene bank: LC732050” [60]. Also, botanical identification was conducted by Dr. Rim Hamdy, a botanist in the Botany Department, Faculty of Science, Cairo University, Giza, Egypt, and a voucher specimen was retained at the Herbarium of the Botany Department (CAI), Faculty of Science. 

Folin–Ciocalteu, 2,2-diphenyl-1-picryl hydrazyl (DPPH), gallic acid standard, sulfuric acid, sodium hydroxide, boric acid, hydrochloric acid, copper sulfate, potassium sulfate, 2,6-dichlorophenol, ascorbic acid, all chemical, reagent, solvent and standards which were used in identification by HPLC were acquired from Sigma-Aldrich, St. Louis, MO, USA.

### 3.2. Estimation of the Chemical Constituents of Dried Stems

**Figure 3:**
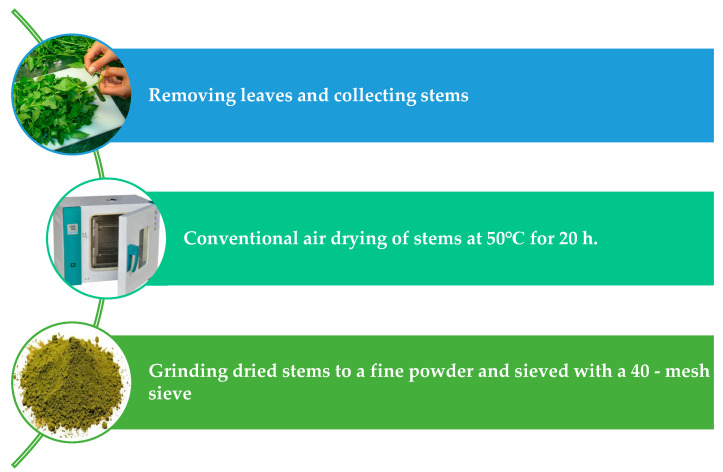
Preparation steps of dried Jew’s mallow stems (DJMS).

The plants were washed and the leaves were separated from the stems. The stems were dried using a conventional dryer at 50 °C for 20 h and ground using the grinder to a fine powder (Touch Elzenoky 40,510 King Kitchen Machine, 1000 Watt, Elzenoky Company, Cairo, Egypt) and sieved with a 40-mesh sieve as shown in Figure 3. The dried samples were stored in polypropylene packages at −18 °C for further analysis. The dried stems’ moisture, ash, fiber, and fat percentages were estimated according to AOAC methods [60]. The protein content was ascertained utilizing the Kjeldahl method. The percentage of protein was determined by multiplying the nitrogen value by a factor of 5.5 [61]. In addition, mineral contents, including Fe, Ca, Mg, P, K and Se were determined according to the method described by AOAC [60], using a Pye Unicom SP1900 atomic absorption spectrometry (Perkin Elmer model 4100ZL; PerkinElmer, Shelton, CT, USA). Carbohydrate was calculated by difference. 

### 3.3. Estimation of Chlorophyll a, b and β-Carotene

The β-carotene and chlorophyll a and b contents were determined using the method described by Youssef et al. [23]. A 200 mg DJMS was vigorously shaken with 10 mL of acetone-hexane combination (4:6) for 5 minutes and filtered through filter paper. The extract volume was precisely modified to 10 mL using a volumetric flask. The extract’s absorbance was measured at 453, 505, 645, and 663 nm wavelength using a spectrophotometer (6505 UV/VIS, Jenway L.T.D., Felsted, Dunmow, UK). β-carotene and chlorophyll a and b contents were determined using the following formulas:

β-carotene (mg 100 mL^−1^) = 0.216 × A663 − 1.220 × A645 − 0.304 × A505 + 0.452 × A453 (1)

Chlorophyll a (mg 100 mL^−1^) = 0.999 × A 663 − 0.0989 × A645 (2)

Chlorophyll b (mg 100 mL^−1^) = −0.328 × A663 + 1.77 × A645, further expressed in mg per 100 g dry weight. (3)

### 3.4. Estimation of Total Phenolic Compounds (TPC)

Methanolic DJMS extract was prepared by maceration of the stem powder (5 mg 100 mL^−1^ methanol 80%). The resulting extract was agitated in a shaker at 300 rpm for 1 h and filtered using Watman No. 1 filter paper. The analysis of all extracts was conducted three times. The TPC of the extract was measured using the Folin–Ciocalteu reagent method as described by El-Beltagi et al. [62], with some modifications. The absorbance was determined at a wavelength of 765 nm utilizing a spectrophotometer. The results were expressed as mg GAE 100 g^−1^ of the sample’s dry weight (DW).

### 3.5. Estimation of Free Radical-Scavenging Activity

The extract solution’s ability to scavenge free radicals was determined following the methodology previously detailed by Yakoub et al. [29], with some modifications. An extract solution (0.1 mL) was added to 3.9 mL DPPH (0.0024 mg 100 mL^−1^ methanol). The solution was stored in a dark environment at room temperature, and the absorbance was quantified at a wavelength of 517 nm after a 30-min incubation period using a UV-visible spectrophotometer. The quantification of free radical scavenging activity was determined as the percentage of inhibition, as defined by the following equation:

(4) $$\mathrm{Scavenging}\;\mathrm{activity}\;\%=\frac{A\;control-A\;sample}{A\;control}\times 100$$

In that order, *A* control and *A* sample represent the absorbance of the DPPH methanolic solution and the DJMS extract.

### 3.6. High-Performance Liquid Chromatography (HPLC) Methods

All HPLC quantification analyses of samples were analyzed in ISO/IEC17025: a 2017 accredited laboratory.

#### 3.6.1. Estimation of Sugar Compounds 

The sample was homogenized using a high-speed homogenizer with deionized water for 3 min, divided into 3 intervals, at a temperature of 60 °C. Subsequently, the homogenized sample was filtered through a membrane with a pore size of 0.22 µm. A portion of 1.5 mL of these solutions was transferred into vials for the analysis. The refractive index detector-coupled Agilent (Series 1200) chromatographic system has a quaternary pump, degasser, and auto-injector. Agilent collected chromatographic data. As previously explained, the samples acquired were analyzed under the conditions specified by Zielinski et al. [63]. The method was verified using the steps followed by Coelho [64]. The technique provided values for recovery (90–106%) and limits of detection (0.03–4.4 g 100 g^−1^) and quantification (0.08–1.99 g 100 g^−1^). 

#### 3.6.2. Estimation of Vitamin B Complex Content 

Vitamin B complexes were fractionated according to the method defined by Antakli et al. [65], with a slight modification using a variable wavelength detector (VWD) instead of a fluorescence detector with the VWD set at 280 nm. This method was validated by following some details, including LOD, LOQ and recovery range (97–100%) [65].

#### 3.6.3. Estimation of Vitamin C Content 

Chromatographic measurements were made using an HPLC system (model Series 1200, Agilent Technologies, Waldbronn, Germany) with a column compartment ODS C18 (250 × 4.6 mm ID, five μm particle size) set at 25 °C. A half gram of the sample and 300 μm of 0.56% (*w*/*v*) meta-phosphoric acid solution were added to the special centrifuge and filtration tube, shaken for 30 s, and centrifuged at 10 °C (10 min, 3000× *g*). The supernatant was filtrated with a 13 mm 0.45 μm Teflon filter disc into a vial for analysis by HPLC. The experiment involves performing an isocratic chromatographic separation using a mobile phase of deionized water/acetic acid (0.1%, *v*/*v*) and MeOH in a relative proportion of 95:5 (*v*/*v*). The eluent flowed at a rate of 0.7 mL min^−1^. Vitamin C was identified by comparing the retention duration of the sample peak with that of the reference ascorbic acid, using a wavelength of 254 nm [66]. The following parameters were determined: limits of detection and quantification (LOQ < 5 μg 100 mL^−1^ and LOD < 2 μg 100 mL^−1^), and recovery (95%) as performed in the same method [66]. 

#### 3.6.4. Estimation of Phenolic and Flavonoid Compounds 

A high-performance liquid chromatography system with a variable wavelength detector (model Series 1200, Agilent Technologies, Waldbronn, Germany) was used. Further, the HPLC was equipped with an autosampler, quaternary pump degasser, and column compartment set at 35 °C. The analyses used a stainless-steel column (4 × 250 mm, i.d.) filled with a C18 reverse phase (BDS 5 μm, Labio, Czech Republic). Samples were prepared using the Schieber et al. method [67] to determine the phenolic acids and flavonoids. The validation parameters are according to the guide for validation set out by da Silva Padilha [68]. The method showed recovery (92–104%) and limits of detection (0.06–0.95 µg 100 g^−1^) and quantification (0.05–1.51 µg 100 g^−1^).

In an ultrasonic bath, DJMS sample weights (100 mg) were extracted in 45 min with 10 mL methanol. The specimens were centrifugated for 7 min at 4200 revolutions per minute. Before analysis, the liquid portion was passed through a Chromafil AO-45/25 polyamide filter and collected in a vial. The HPLC procedure commenced with a linear gradient at a fluid flow of 1 mL min^−1^, utilizing a mobile phase consisting of water/acetic acid (98:2 *v*/*v*, referred to as solvent A) and acetonitrile/methanol (50:50 *v*/*v*, referred to as solvent B). The gradient began with 5% solvent B and progressively increased to 30% at 25 min, 40% at 35 min, 52% at 40 min, 70% at 50 min, and finally reached 100% at 55 min. A 5-min wash in both solvents restored the starting conditions. All chromatograms were displayed at 280 nm and flavonoids at 330 to determine phenolic acids. All components were discovered and measured by comparing the peak areas with external standards.

#### 3.6.5. Estimation of Isoflavone Compounds 

Extraction was carried out as described by Kaufman et al. [69]. The validation parameters are according to the guide for validation set out by da Silva Padilha [68], and showed recovery (97–102%) and limits of detection (0.06–0.13 µg 100 g^−1^) and quantification (0.06–0.29 µg 100 g^−1^). Half a gram of the samples was prepared and added to a test tube containing 4 mL of 80% MeOH. These samples were vortexed, and the supernatant was collected and placed in 15 mL CorexTM centrifuge tubes. The previous step was repeated three times for each sample. Tubes were centrifuged for 20 min at 27,000× *g* at 22 °C in a Superspeed refrigerated centrifuge. The supernatant, which contained the isoflavonoids, was then air-dried. Next, 1 mL of 80% MeOH was added to the air-dried test tubes. The tubes were then vortexed, covered, and kept in a refrigerator at 4 °C until high-performance liquid chromatography analysis. HPLC was performed using a hypersil BDS C18 column (4.6 × 250 mm in size) at a 254 nm wavelength.

### 3.7. Cytotoxicity Assay

Huh-7: liver cancer and MDA-MB-231: breast cancer (transitional cell carcinoma) were obtained from Nawah Scientific Inc. (Mokatam, Cairo, Egypt). Cells were maintained in McCoy’s media supplemented with 100 mg mL^−1^ of streptomycin, 100 units mL^−1^ of penicillin, and 10% of heat-inactivated fetal bovine serum in a humidified, 5% (*v*/*v*) CO_2_ atmosphere at 37 °C. Cell viability was evaluated using the SRB test. The 100 μL aliquots of cell suspension containing 5 × 10^3^ cells were placed in 96-well plates and cultured in a complete medium for 24 h. The cells were subjected to an additional 100 μL of medium-containing DJMS extract at different doses, ranging from 0.01 to 1000 μg/mL. After 72 h of DIMS extract treatment, the cells were immobilized by replacing the medium with 150 μL of 10% TCA and incubating them at 4 °C for 1 h. The TCA solution was eliminated, and the cells were rinsed five times with distilled water. A total of 70 microliter portions of a 0.4% weight/volume solution of SRB were added and placed in a dark environment at room temperature for 10 min. The plates were rinsed three times with a 1% acetic acid solution and left to dry naturally overnight. Next, 150 μL of TRIS (10 mM) solution was introduced to dissolve the SRB stain bound to the protein. The solution’s absorbance was subsequently measured at a wavelength of 540 nm using a BMG LABTECH^®^-FLUO star Omega microplate reader (Ortenberg, Germany) [70,71]. The cytotoxicity % was calculated using the provided equation and corrected absorbance.

Cytotoxicity % = (100 × (control − sample)(5)

### 3.8. Statistical Analysis

Costat Version 6.45 was used for all statistical analysis (CoHort Software Version 6.45, Monterey, CA, USA). One-way analysis of variance (ANOVA) was used as the primary statistical analysis method. A Shapiro–Wilk test was used to verify that each experiment’s normality distributions were correct. A Duncan’s multiple range test was performed with a 5% significant point. A Bartlett’s test was employed to determine whether the variation within sample groups was homogeneous. Systat Software Inc., Erkrath, Germany, used SigmaPlot Version 12 to create the graphs and figures.

## 4. Conclusions

The stem of *C. olitorius* is highly regarded for its substantial nutritional content, which includes abundant dietary fiber, protein, vitamins (B-complex), and minerals (iron). Moreover, it contains various major phytoconstituents, namely phenolic, flavonoid, and isoflavone compounds (ellagic acid, ferulic acid, chlorogenic acid, luteolin—7-*O*-glucoside, naringin, apigenin—6—rhamnose—8 glucoside, isorhamnetin, and genistein), vitamin C, and chlorophyll a and b, which give it high potential as a natural antioxidant. The data presented here align with earlier findings of *C. olitorius leaves* regarding their biological activity. Overall, the dried stems are characterized by being edible and available in large quantities as a cheap source of bioactive compounds. Moreover, the utilization of food industry waste consists of sustainability and environmental protection policies. Therefore, according to the results of this study on the phytoconstituents and biological activities of *C. olitorius* dried stem, it shows promise as a viable alternative ingredient in the pharmaceutical and nutraceutical industries, especially for cancer. Otherwise, additional in vitro and in vivo studies are necessary to comprehend how the natural chemical may serve as a possible anticancer medication.

## Figures and Tables

**Table 1:** Identification of sugar types and vitamin and mineral contents of dried Jew’s mallow stems.

Sugar Types	Unit (g 100 g^−1^)	Vitamins	Unit (mg kg^−1^)	Minerals	Unit (μg kg^−1^)
Stachyose	6.25	Thiamin (B1)	5.62	Fe	225.20
Sucrose	9.23	Riboflavin (B2)	67.52	Ca	1.85
Maltose	0.59	Pyridoxine (B6)	9.31	Mg	0.83
Galacturonic	0.52	Folic (B9)	25.68	P	0.21
Glucose	0.62	Cobalamin (B12)	146.80	K	2.12
Xylose	0.27	Vitamin C	6.49	Se	<0.20
Sorbose	0.20				
Galactose	0.39				
Rhamnose	0.93				
Mannose	0.97				
Fructose	0.46				
Arabinose	0.06				
Mannitol	0.01				
Ribose	0.01				

**Table 2:** Identifying phenolic, flavonoid and isoflavone compounds (µg 100 g^−1^) of dried Jew’s mallow stems.

Phenolic	Concentration	Flavonoids	Concentration	Isoflavones	Concentration
Pyrogallol	2785.25	Apigenin–6—arabinose—8 galactoside	2747.54	Isorhamnetin	5502
Gallic acid	115.07	Apigenin—6—rhamnose—8 glucoside	3078.87	Daidzein	59.91
3-hydroxytyrosol	50.52	Rutin	1944.6	Genistein	34.96
Catechol	394.82	Naringin	4296.94	Isoorientin	57.57
p-aminobenzoic acid	338.67	Luteolin-7-*O*-glucoside	4314.48	Biochanin	1.77
Rosmarinic acid	2538.82	Catechin	1787.88		
Chlorogenic acid	3757.08	Quercetin	1744.11		
p-hydroxybenzoic acid	586.76	Apigenin-7-glucoside	790.23		
Benzoic acid	403.47	Quercetin-3-glucoside	1791.38		
Caffeic acid	359.08	Naringenin	691.14		
Vanillic acid	345.66	Kaempferol 3-(2-p-coumaroyl) glucoside	1780.45		
Caffeine	903.28	Kaempferol	1585.73		
Ferulic acid	3628.29	Apigenin	868.74		
Ellagic acid	4905.26				
Salicylic acid	706.69				
Coumarin	135.28				

## Data Availability

Data are contained within the article.
